# Public policy interventions to mitigate household food insecurity in Canada: a systematic review

**DOI:** 10.1017/S1368980024000120

**Published:** 2024-01-15

**Authors:** Leanne Idzerda, Tricia Corrin, Calin Lazarescu, Alix Couture, Eric Vallières, Sara Khan, Valerie Tarasuk, Lynn McIntyre, Alejandra Jaramillo Garcia

**Affiliations:** 1 Centre for Surveillance and Applied Research, Public Health Agency of Canada, Ottawa, Ontario, Canada; 2 Scientific Operations and Response, Public Health Agency of Canada, Guelph, Ontario, Canada; 3 Regional Operations, Public Health Agency of Canada, Montreal, Quebec, Canada; 4 Environmental Health Science and Research Bureau, Health Canada, Toronto, Ontario, Canada; 5 Department of Nutritional Sciences, University of Toronto, Toronto, Ontario, Canada; 6 Cumming School of Medicine, University of Calgary, Calgary, Alberta, Canada

**Keywords:** Food insecurity, Public policy interventions, Systematic review

## Abstract

**Objective:**

The objective of this systematic review is to synthesise the evidence on public policy interventions and their ability to reduce household food insecurity (HFI) in Canada.

**Design:**

Four databases were searched up to October 2023. Only studies that reported on public policy interventions that might reduce HFI were included, regardless of whether that was the primary purpose of the study. Title and abstract screening, full-text screening, data extraction, risk of bias and certainty of the evidence assessments were conducted by two reviewers.

**Results:**

Seventeen relevant studies covering three intervention categories were included: income supplementation, housing assistance programmes and food retailer subsidies. Income supplementation had a positive effect on reducing HFI with a moderate to high level of certainty. Housing assistance programmes and food retailer studies may have little to no effect on HFI; however, there is low certainty in the evidence that could change as evidence emerges.

**Conclusion:**

The evidence suggests that income supplementation likely reduces HFI for low-income Canadians. Many questions remain in terms of how to optimise this intervention and additional high-quality studies are still needed.

Household food insecurity (HFI) is an important indicator of material deprivation and a serious chronic public health issue that affects, from the 2021 Canadian Income Survey, 18·4 % of Canadian households^(1)^. Household food security is monitored in Canada using the Household Food Security Survey module^(2)^, whose questions are premised on a household’s financial ability to access adequate food. As such, food insecurity can be defined as the inadequate or insecure access to food due to financial constraints^(3)^.

HFI has substantial adverse impacts on individuals’ health and the related healthcare costs in Canada^(4,5)^. People living in food insecure households have poorer self-rated mental, physical and oral health, greater stress and are more likely to suffer from chronic conditions such as diabetes, hypertension and mood or anxiety disorders^(6–8)^.

The persistently high prevalence and negative health implications of HFI have raised the spectre of the role of social protection programmes such as social assistance benefits, employment insurance benefits, universal childcare benefits and housing subsidies in mitigating households’ economic circumstances leading to HFI. Although tightly linked to income, HFI also reflects a household’s broader material circumstances including owning assets such as property, income stability and debt^(9)^. The measurement of HFI during the COVID-19 pandemic was hampered by the interruption of survey data collection, but as more comparable data have emerged, food insecurity rates in high-income countries appear to have remained relatively stable through the pandemic^(1,10)^, in part perhaps because COVID monetary benefits mitigated the pandemic’s major income shock^(11)^. In Canada, the USA and Australia, pandemic recovery has been associated with increased food insecurity, possibly because inflation and food prices increases have pushed more economically vulnerable households into a food insecure state^(1,12,13)^. Thus, there is a need to identify interventions that might mitigate households’ economic vulnerability to food insecurity. This systematic review (SR) aims to synthesise the evidence on the impact of public policy interventions aimed at improving household financial circumstances on HFI in Canada. Public policy interventions refer to state-level sponsored programmes or activities at any level of government. Food-based interventions that seek to directly respond to households’ food needs were specifically excluded.

## Methods

This SR was guided by the Cochrane Handbook for Systematic Reviews^(14)^ and follows the PRISMA reporting guidelines^(15)^. The original research question was: ‘*What interventions are effective in reducing household food insecurity in Canada?’* The protocol was created *a priori* and registered in Prospero (CRD42021254450).

During the SR process and in discussions with experts in the field of food insecurity (VT, LM), it became clear that the interventions should be grouped into two categories: public policy interventions (e.g. income support, housing assistance programmes) and food-based interventions (e.g. food banks, gardening programmes). These two types of interventions work at different levels. Food-based interventions endeavour to address food shortages at the household level directly, whereas public policy interventions target the underlying economic vulnerability of households to a range of basic needs including food but also housing and employment supports.

The analysis and reporting were conducted separately for the two types of interventions, resulting in two SR. This SR attempts to answer the question ‘*What public policy interventions are effective in reducing household food insecurity in Canada’.* The results of food-based interventions will be reported in a separate SR (Idzerda et al, Unpublished, 2024). The eligibility criteria, search strategy and study selection described below detail the SR process for the original research question, whereas the data extraction, risk of bias and Grading of Recommendations, Assessment, Development and Evaluation (GRADE) are specific to the SR on public policy interventions.

### Eligibility criteria

Primary studies in English or French that assessed an intervention affecting households in Canada, had a comparison group and measured an outcome of HFI were included. The full lists of inclusion and exclusion criteria are included in Supplementary Material A.

### Search strategy

The search strategy was developed by a Health Canada research librarian in collaboration with the authors (Supplementary Material B). It underwent a Peer Review of Electronic Search Strategies and was reviewed for quality by a second independent librarian^(16)^. The original search was implemented in April 2021, updated in November 2022 with a final update on 5 October 2023. Four electronic bibliographic databases were searched: Scopus, OVID Medline, Embase and EconLit. A complementary grey literature search was conducted in June 2021. Finally, the reference lists of seventeen related reviews were searched and experts were consulted to ensure that all eligible articles were included.

### Study selection

To verify potential eligibility, all titles and abstracts were screened by two independent reviewers using a standardised form developed *a priori*, which was piloted by all reviewers (LI, TC, CL, AC, EV, SK) in the software DistillerSR Version 2.37^(17)^. Next, two reviewers independently screened the full text of each potentially eligible article. The reasons for excluding a study were recorded in both stages of screening. A list of excluded studies is available in Supplementary Material C.

All conflicts were resolved by consensus or a third reviewer. This was also done for data extraction, risk of bias and GRADE.

### Data extraction

A data extraction form was developed *a priori* and was piloted by all reviewers. Two reviewers independently extracted data for each included study. Study information (objectives, study design, time of data collection, description of intervention and the method or tool to measure food insecurity) and participant characteristics (including any subgroups of interest) were extracted for all studies. The outcome of interest was change in level of food insecurity (food secure, marginal, moderate or severe) over time.

### Data analysis

Where studies presented results using the same dataset, the study with the longest follow-up period was selected. All data points were utilised if there was no overlap in the data (i.e. all years reported in population level surveys). For studies with monetary interventions, dollar values reported were standardised to 2023 using the Bank of Canada Inflation Calculator (September 2023 Consumer Price Index)^(18)^.

Data were synthesised narratively, as heterogeneity in the interventions meant that the data were not appropriate for pooling.

### Risk of bias

The risk of bias was determined using the Effective Public Health Practice Project Quality Assessment Tool (EPHPP)^(19)^. This tool was deemed the most appropriate and encompassing due to the wide variety of study types. Two reviewers independently rated the risk of bias for each study.

### Certainty of the evidence

The GRADE framework was used to rate the certainty and strength of the body of evidence^(20)^. Each outcome was assessed independently by two reviewers. The GRADE decision rules as they were applied to this study are presented in Supplementary Materials D. Randomised controlled trials and large population-based studies were started at high certainty of evidence, whereas observational studies were started at low certainty of evidence.

## Results

Three categories of public policy interventions were found and assessed: income supplementation, housing assistance programmes and food retailer subsidies.

### Descriptive summary of included studies

**Fig. 1:**
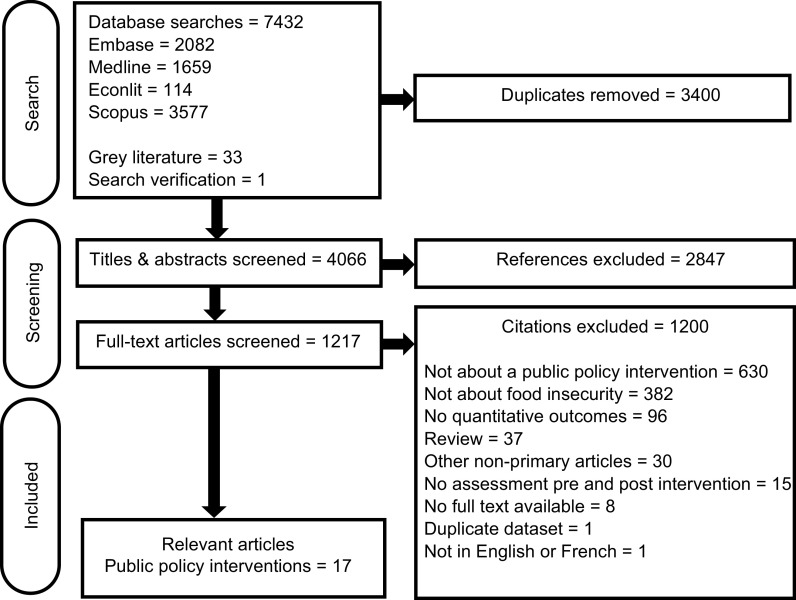
PRISMA flow diagram of articles through the systematic review process

Of the 7,432 references screened for eligibility, seventeen reported on public policy interventions to reduce HFI (Fig. 1). Study characteristics are presented in Table 1. Sixteen (94 %) of the articles were published in the last 10 years (since 2013).

**Table 1:** Study characteristics of included studies

Author, publication year	Location	Implementer	Intervention	Year and benefit amount from text	Standardised to 2023	Study Design	Dataset	Year of data collection	Population	Comparator	Total sample size	Overall risk of bias rating*
**Income supplementation**												
Brown, 2019	National (excluding Ontario, Newfoundland and Labrador and the Territories)	Federal policy intervention	CCB	2016: $6,800	$8,368	Natural policy experiment (repeated cross-sectional)	CCHS	2015–2018	Households with children under 18	Households without children under 18	14 712	Low
Emery, 2013A	National (excluding Prince Edward Island and the Territories)	Federal policy intervention	OAS and GIS	2009: $13 700 annual maximum	$18 932	Natural policy experiment (repeated cross-sectional)	CCHS	2007–2008	Single low-income adults (aged 65–69) who are eligible for public pension benefits	Single low-income adults (aged 60–64) who are ineligible for public pension benefits	302 835 (population weighted)	Low
Emery, 2013B	National (excluding the Territories)	Federal policy intervention	OAS and GIS	2011: $14 708 annual maximum	$19 330	Natural policy experiment (repeated cross-sectional)	CCHS	2009–2010	Single low-income adults (aged 65+) who are eligible for public pension benefits	Single low-income adults (aged 55–64) who are ineligible for public pension benefits	500 000 (population weighted)	Low
Ionescu-Ittu, 2014	Quebec, British Columbia, Alberta, Nova Scotia, Nunavut, Northwest Territories	Federal policy intervention	UCCB	2006: $1,662 true effect on income	$2,412	Natural policy experiment (repeated cross-sectional)	CCHS	2001–2009	Individuals over 12 years old living in households with children under 6 years old	Individuals over 12 years old living in households with children aged 6–11 but no children under 6 years old	40 501	Low
Li, 2016	British Columbia	Provincial policy intervention	Welfare	2007: $293–$1,851 increase in welfare *v*. 2005 reference year†	$381–$2,405	Natural policy experiment (repeated cross-sectional)	CCHS	2005–2012	Residents of British Columbia receiving welfare payments (for welfare increase intervention)	Residents of British Columbia not receiving welfare payments	58 656	Low
Loopstra, 2015	Newfoundland and Labrador	Provincial policy intervention	Provincial income support payments	Data or reference not provided		Natural policy experiment (repeated cross-sectional)	CCHS	2007–2012	Population receiving income support payments	Population prior to receiving income support payments	11 239	Low
Men, 2023A	National (excluding the Territories)	Federal policy intervention	CCB	2019: $724 annual average	$824	Natural policy experiment (repeated cross-sectional)	CIS	2018–2020	Households with children under 6 years	Households with children aged 6–17	28 435	Low
Men, 2023B	National (excluding the Territories)	Federal policy intervention	EI	2019: $8794 ± 6670 annual average	$10 182 ± $7,722	Natural policy experiment (repeated cross-sectional)	CIS	2018–2019	Unemployed workers receiving EI	Unemployed workers not receiving EI	4,085	Low
McIntyre, 2016	National (excluding Prince Edward Island and the Territories)	Federal policy intervention	OAS and GIS	2015: $15 950 annual minimum	$19 890	Natural policy experiment (repeated cross-sectional)	CCHS	2007–2013	Unattached low-income adults (aged 65–74) who are eligible for public pension benefits	Unattached low-income adults (aged 55–64) who are ineligible for public pension benefits	8,019	Low
Tarasuk, 2019	Ontario	Provincial policy intervention	OCB	2007–2008: $649 ($889); 2009–2010: $1,368 ($1,855); 2011–2012: $1,377 ($1,789); 2013–2014: $1,837 ($2,315) increase from 2005–2006 reference year		Natural policy experiment (repeated cross-sectional)	CCHS	2005–2014	Households with children eligible to receive the Ontario Child Benefit	Households (single or families) not eligible to receive the Ontario Child Benefit	9,139	Low
**Housing interventions**												
Kirkpatrick, 2011	Toronto, Ontario	Provincial policy intervention	Subsidised housing	Unspecified		Cross-sectional	Data collected by the authors for the purpose of this study – HFSSM	2005–2007	Low-income families residing in high-poverty urban neighbourhoods (subsidised-rent households)	Low-income families residing in high-poverty urban neighbourhoods (market-rent households)	473	Moderate
Li, 2016	British Columbia	Provincial policy intervention	Rental assistance	2006: $4,548 per family annually	$6,601	Natural policy experiment (repeated cross-sectional)	CCHS	2005–2012	Renter households (for rental assistance intervention)	Homeowners	58 656	Low
Lachaud, 2020	Toronto, Ontario	Community-based intervention	Housing assistance (unspecified)	Unspecified		Randomised controlled trial	Data collected by the authors for the purpose of this study - modified HFSSM	Baseline: 2009–2011 Follow-up: 2 years after baseline	Homeless adults or precariously housed adults with a mental disorder, who received housing assistance	Homeless adults or precariously housed adults with a mental disorder, who did not receive housing assistance	575	High
Loopstra, 2013	Toronto, Ontario	Provincial policy intervention	Subsidised housing (unspecified)	Unspecified		Longitudinal study	Data collected by the authors for the purpose of this study – modified HFSSM	2005–2008	Low-income families residing in high-poverty urban neighbourhoods (subsidised-rent households)	Low-income families residing in high-poverty urban neighbourhoods (market-rent households)	331	Moderate
McIntyre, 2017	National (excluding Prince Edward Island, New Brunswick and the Territories)	Federal policy intervention	Home ownership	NA		Natural policy experiment (repeated cross-sectional)	CCHS	2007–2010	Households post-recession	Households pre-recession	139 600	Low
O’Campo, 2017	Vancouver, British Columbia; Winnipeg, Manitoba, Toronto, Ontario, Montreal, Quebec and Moncton, New Brunswick	Community-based intervention	Housing assistance (varies by city)	Varies by city		Randomised controlled trial	Data collected by the authors for the purpose of this study - modified HFSSM	2009–2011	Homeless adults or precariously housed adults with a mental disorder, who received housing assistance	Homeless adults or precariously housed adults with a mental disorder, who did not receive housing assistance	2,097	High
Pankratz, 2017	Waterloo, Ontario	Community-based intervention	Housing assistance	2014: $4,200	$5,292	Quasi-experimental (pre–post design with control)	Data collected by the authors for the purpose of this study – modified HFSSM	Baseline: 2014 Follow-up: 6 months after baseline	People experiencing chronic homelessness in Waterloo region and receiving home support *plus* housing assistance	People experiencing chronic homelessness in Waterloo region and receiving home support only (no housing assistance)	60	High
**Food retail subsidy interventions**												
St-Germain, 2019	Nunavut	Federal policy intervention	Nutrition North Canada	Varies by community		Natural policy experiment (repeated cross-sectional)	CCHS	2007–2016	Households living in Nutrition North Canada eligible communities prior to programme introduction	Households living in Nutrition North Canada eligible communities after the programme introduction	3,250	High

CCB, Canada Child Benefit; OAS, Old Age Security; GIS, Guaranteed Income Supplement; UCCB, Universal Child Care Benefit; OCB, Ontario Child Benefit; EI, Employment Insurance; CCHS, Canadian Community Health Survey; HFSSM, Household Food Security Survey Module; CIS, Canadian Income Survey; EI, Employment Insurance.

* Domain level risk of bias results for all studies can be found in Supplementary Material E.

† Text only indicates an up to 11.7 % increase in welfare income. Difference from reference year calculated from Tweddle, A., Battle, K., Torjman, S.: Welfare in Canada 2012. Values presented in standardised to 2012.

### Risk of bias assessments

Overall, income supplementation studies were at low risk of bias, while housing and food retail subsidy intervention studies were at moderate to high risk of bias (Table 1). A summary of the detailed risk of bias results for all studies can be found in Supplementary Material E.

### Summary of findings

#### Income supplementation

Ten studies on the impact of income supplementation on HFI were identified^(21–30)^. This included direct payments from a government body to an individual or household, such as child care benefits, guaranteed income supplementation for seniors, employment insurance and social assistance. Three of these reported on the same data for the sample population^(23–25)^. Only the data from one of the three studies were used^(23)^ as this study had the longest follow-up and encompassed the data from both other papers^(24,25)^.

Among low-income populations, three studies demonstrated that income supplementation interventions had a positive effect on reducing moderate and severe HFI with a high level of certainty (Table 2). The odds of HFI were lowest in the intervention with the highest dollar value ($19 890/year) of income supplementation (OR 0·30, 95 % CI 0·27–0·33)^(23)^ and increased (OR 0·85, 95 % CI 0·75, 0·96) as the dollar value ($8,368/year) decreased^(21)^. A similar but less pronounced trend was observed in five studies reporting on low-income households experiencing marginal, moderate or severe food insecurity. The lowest dollar value ($824) had no impact on reducing food insecurity^(29)^, however as the dollar amount increased so did the associated adjusted OR, becoming significant at higher levels of income supplementation. In addition, three studies also assessed the change over time against a matched control group (e.g. difference in difference analysis). In all three studies, the control group saw no change over time, while the intervention group had reductions in HFI over time^(21,28,30)^.

**Table 2.** Summary of findings Table for income supplementation interventions

	Studies				Number of participants		Effect size				
Intervention type	Author, year	Study design	Population	Outcome	Income supplementation	No income supplementation	OR	95 % CI	Benefit amount^*^	Direction of effect	Certainty
Income supplementation for low income households	Brown, 2019	Natural policy experiment (repeated cross-sectional)	Low-income	Moderate and severe HFI	41 455^†^		0·85	0·75, 0·96	$8,368	Favours supplementation	High^2^
	Li, 2016				1,217	36 787	2007: 0·67	0·47, 0·96	$381 - $2,405		
	McIntyre, 2016				3,498	4,521	0·30	0·27, 0·33	$19 890		
	Brown, 2019	Natural policy experiment (repeated cross-sectional)	Low-income	Total HFI (marginal, moderate and severe)	7,579^†^		0·91	0·81, 1·03	$8,368	Favours supplementation	Moderate^2,3^
	Li, 2016				1,217	36 787	2007: 0·67	0·48, 0·94	$381 - $2,405		
	Loopstra, 2015				719	10 520	2008: 0·95	0·75, 1·20	Data not provided		
							2009: 0·76	0·59, 0·98			
							2010: 0·74	0·56, 0·98			
							2011: 0·70	0·53, 0·92			
							2012: 0·96	0·70, 1·31			
	Men, 2023A				11 025^†^		0·31	0·17, 2·17	$824		
	Men, 2023B				3,200^†^		0·77	0·63, 0·89	$10 182 ± $7,722		
	Loopstra, 2013	Longitudinal study			331		A gain of $2000 in household income was associated with a decrease of 0·29 in reported number of affirmed responses on the HFSSM		$2,740		
Universal Income supplementation	Brown, 2019	Natural policy experiment (repeated cross-sectional)	General population	Moderate and severe HFI	14 712^†^		0·90	0·84, 0·97	$8,368	Favours supplementation	Moderate^1,2,3^
	Ionescu-Ittu, 2015				6,542	16 195	0·29	0·27, 0·32	$2,412		
	Men, 2023A				28 435^†^		0·66	0·35, 4·76	$824		
	Brown, 2019	Natural policy experiment (repeated cross-sectional)	General population	Total HFI (marginal, moderate and severe)	14 712^†^		0·96	0·90, 1·02	$8,368	Favours supplementation	Moderate^2,3^
	Men, 2023A				28 435^†^		0·35	0·27–0·74	$824		
	Men, 2023B				4,390^†^		0·77	0·66, 0·90	$10 182 ± $7,722		
	Tarasuk, 2019				9,139^‡^		2007–2008: 0·85	0·69, 1·04	$889		
							2009–2010: 0·73	0·59, 0·91	$1,855		
							2011–2012: 0·66	0·53, 0·82	$1,789		
							2013–2014: 0·83	0·65, 1·06	$2,315		

* All dollar values are standardised to 2023.

† Values not reported by exposure group.

‡ Tarasuk *et al*. (2019) provides prevalence and odds ratios of food insecurity in relation to survey cycles. Only total number of participants was extracted as food insecurity prevalence changes per survey cycle.

List of abbreviations: OR: Odds Ratio, CI: Confidence Interval

Grade reasoning:

1 Inconsistency: differences in effect estimate among studies.

2 Indirectness: study population not representative of the whole population.

3 Imprecision: OIS value not met, or no effect/not significant effect with large confidence intervals.

Among the general population, there was a mostly positive effect of exposure to federal or provincial child benefit programmes on HFI as demonstrated across four studies with moderate certainty (Table 2). Assessing the impact of benefits on moderate and severe HFI among the general population in three studies revealed the same relationship, except for one study^(29)^. The reason for this may be associated with the low dollar value associated with the intervention.

#### Housing assistance programmes

The effect of housing assistance programmes on HFI was assessed in seven studies, Table 2 ^(28,31–36)^. Housing assistance programmes provide cash benefits designated for rental or other housing costs in approved commercial or public housing settings. This includes housing for precariously housed individuals, subsidised housing (reduced cost of public housing) and rental assistance programmes (money given to low-income households to use towards rental costs). Three studies assessed the impact of housing assistance programmes on HFI in homeless or precariously housed individuals and found no effect except for one subgroup where a larger proportion of those with high mental health needs achieved food security following the intervention *v*. those who did not receive the housing intervention^(31–33)^.

The impact of exposure to subsidised housing programmes on HFI among low-income populations was evaluated in three studies, Table 3 ^(28,35,36)^. Two studies found no association between low-income families that received housing subsidies and HFI in the large city of Toronto^(35,36)^. However, the odds of HFI were lower among families with subsidised rent compared to households with market rent on a waitlist for subsidised housing (OR = 0·51; 95 % CI 0·30–0·86)^(36)^. In the third study, there was no reduction in HFI following the introduction of a rental assistance programme ($550 per month, standardised to 2023 values) in the Province of British Columbia^(28)^. All post-intervention follow-up periods looking at HFI levels were at least 6 months in length.

**Table 3.** Summary of findings Table for housing interventions

Intervention type	Author, year	Study design	Population	Outcome	Housing assistance	No housing assistance	Effect size	Direction of effect	Certainty
Housing assistance programmes	Pankratz, 2017; Lachaud, 2020; O’Campo, 2017	Cohort (two groups pre + post) Randomised controlled trial	Homeless and precariously housed individuals	Moderate and severe HFI	997	772	No effect	NA	Low^1,3,4^
	O’Campo, 2017	Randomised controlled trial	Homeless and precariously housed individuals – high needs mental health	Moderate and severe HFI	469	481	61 % of intervention group *v*. 54 % of control group achieved food security (*P* = 0·02).	Favours housing assistance	Low^1,3,4^
Subsidised housing programmes	Loopstra, 2013	Cohort-analytic (pre–post study)	Low-income	Marginal, moderate and severe HFI	186	145	No effect	NA	Very low^1,2,3,4^
	Kirkpatrick, 2011	Cross-sectional	Low-income	Moderate and severe HFI	251	222	(1) No difference in HFI for subsidised rent *v*. market rent households (2) HFI lower among market rent households *v*. those on the list for subsidised rent OR = 0·51 (95 % CI 0·30–0·86)	NA Favours subsidised housing over those on waitlist for subsidised housing	Very low^1 2,3,4^
	Li, 2016	Repeated cross-sectional	Low-income	Marginal, moderate and severe HFI	1,217	36 787	No effect	NA	Very low^1,2,3,4^
Homeownership	McIntyre, 2017	Repeated cross-sectional	General population	Moderate and severe HFI	17 926	68 050	OR = 1·16, 95 % CI 1·05–1·29	Renter’s risk of food insecurity increased significantly post-recession	Moderate^3^

Grade reasoning:

1 Study limitations: study had high or moderate risk of bias.

2 Inconsistency: differences in effect estimate among studies.

3 Indirectness: study population not representative of the whole population.

4 Imprecision: OIS value not met, or no effect/not significant effect with large confidence intervals.

Overall, these studies showed, with low to very low certainty, that housing assistance programmes for homeless or precariously housed individuals, and housing subsidies for low-income populations, may have little to no effect on HFI.

A study on the impact of home ownership on HFI in Canada before and after the 2008–2009 recession demonstrated that home ownership likely reduced the risk of HFI during this time^(34)^. Specifically, among renters, the risk of HFI increased significantly post-recession (OR = 1·16, 95 % CI 1·05–1·29), whereas homeowners had a non-significant slight increase in HFI over the same period^(34)^.

#### Food retailer subsidy interventions

Food retailer subsidy programmes include direct payments by government bodies to food retailers to reduce the price of foods sold to the public prior to the point of purchase. One study assessed a federal food retailer subsidy programme, Nutrition North Canada, in Nunavut Territory, Table 4 ^(37)^. After controlling for several covariates, the rate of HFI increased by 13·2 percentage points (95 % CI 1·7–24·7) after implementation of the subsidy programme^(37)^. The implementation of Nutrition North Canada may have increased rates of HFI, but the evidence is very uncertain.

**Table 4.** Summary of findings Table for food retail interventions

Intervention Type	Author, year	Study Design	Population	Outcome	After food retail subsidy	Before food retail subsidy	Effect size	Direction of effect	Certainty
Market Food Subsidy	St. Germain, 2019	Natural-policy experiment	General population	Marginal, moderate and severe HFI	900	1360	Food insecurity increased by 13·2 percentage points (95 % CI 1·7–24·7) after the full implementation of the subsidy programme	Favours pre-market subsidy	Low^1,2^

Grade reasoning:

1 Study limitations: study had high or moderate risk of bias.

2 Indirectness: study population not representative of the whole population.

## Discussion

The objective of this SR was to synthesise the evidence on public policy interventions to mitigate HFI in Canada. Three categories of interventions were found and assessed: income supplementation, housing support and food retailer subsidies.

### Income supplementation studies

This SR found that income supplementation (in the range of $824–$19 890 CDN standardised to 2023) for low-income Canadians likely leads to fewer households being food insecure, with the size of effect possibly increasing as the monetary value of the intervention increases. This is aligned with another recent SR conducted in Canada and the USA, which found moderate-certainty evidence of an association between offering monetary assistance and reduced food insecurity (ten studies; pooled random effects; adjusted OR, 0·64; 95 % CI 0·49–0·84)^(38)^. Although the studies were generally well executed, income supplementation has been inferred from an exposure and was never actually observed. In the absence of experimental data, such as data from a basic income experiment, it will be difficult to determine the ‘dose’ of income necessary to mitigate HFI in vulnerable households. Future research on a possible dose–response curve should be undertaken to set the threshold for which income supplementation has a meaningful impact on HFI in Canada.

A limitation of the income supplementation studies is that most utilised the income variable of the Canadian Community Health Survey (CCHS). This was self-reported, before-tax and imputed by Statistics Canada for 30 % of respondents. In some, but not all, cases the imputation was considered in the analysis. It is likely that there is measurement error on this variable resulting in misclassification of low-income individuals. Additionally, there are marked differences between studies in the operational definitions of what has here been referred to as ‘low income’. This heterogeneity matters because the sensitivity of HFI to income interventions is likely greatest among the most resource-constrained households, but it has not been feasible to take baseline incomes into account in this analysis. Comparison of these studies is further limited as the adjusted OR drawn from studies differed in their identification of and adjustments for potentially confounding factors and observed the effects of the increments in income over different periods of time. Whether the initial effects of increases in benefits on HFI are sustained over time depends on several factors including changes in macroeconomic conditions and policy context (e.g. whether new benefits are indexed to inflation or how their introduction affects other relevant programmes and policies). Since the CCHS and Canadian Income Survey exclude people living in remote locations and on First Nation reserves, as well as those within institutions, the results of this SR cannot be applied to those populations.

An additional limitation has been the inability to determine whether the effects observed here represent reductions in the likelihood or severity of HFI among already-affected households, or the prevention of HFI (or more severe food insecurity). The studies reviewed all used cross-sectional survey data that included measures of HFI over the prior 12 months. Within-household changes were not observed over time; rather, inferences about the effectiveness of specific interventions were drawn from comparisons of HFI status among comparable groups before and after the introduction of policy changes. Although some studies employed robust cross-sectional designs that utilise econometric methods, which capitalise on natural variations in policies to estimate an intent-to-treat^(21,22,37)^, longitudinal studies may be useful to distinguish interventions that prevent food insecurity from those that reduce its prevalence or severity among already-affected households.

### Housing assistance studies

This SR showed that housing assistance programmes for homeless and precariously housed populations as well as housing subsidies for low-income populations may have little to no effect on HFI. The main limitation is the small number and limited scope of the included studies. More high-quality experimental studies among different population groups (low-income, precariously housed and homeless) across the full spectrum of existing policy interventions that potentially impact households’ housing circumstances (e.g. rent supplements, rent controls and rent-geared-to-income housing) are required before one can determine whether there is an impact of these policies on HFI. In studies of effectiveness, it is also important to consider jurisdictional differences in housing policies. Home ownership, compared to renting, seemed to be protective against HFI in one study. Therefore, high quality experimental studies focused on affordable home ownership should also be explored, particularly since both provincial and federal governments in Canada incentivise home ownership^(39)^.

### Food retailer subsidy study

Assessment of a single study on exposure to the Nutrition North Canada food retailer subsidy revealed that HFI increased following the introduction of the programme among remote northern populations. Nunavut, the territory studied, has long been characterised by much higher rates of food insecurity than the rest of Canada^(37)^. Whether the observed increase was directly related to the introduction of the food retailer subsidy programme cannot be established, and there has been no research to determine whether food insecurity similarly increased in other areas covered by the programme. Nonetheless, the persistently high rates of food insecurity in northern Canada have brought this programme under review by deferral authorities^(40)^.

There is also a clear need for effective tailored interventions to mitigate food insecurity among Indigenous communities and in northern Canada. As Nutrition North Canada’s implementation possibly led to worsening food insecurity in at least one target area, a place to start is to rethink what changed with the introduction of this programme, in consultation with Indigenous communities, recognising that income and Indigenous food sovereignty are important considerations^(41)^.

This SR found studies concerning three categories of public policy interventions. It is important to note that no literature was found for some categories of public policy interventions, such as studies evaluating the effects of other market subsidy interventions (e.g. programmes that subsidise out-of-pocket costs for essentials such as utilities, prescription drugs and dental care). Further original studies on the other types of public policy interventions should be conducted.

Although Canada monitors HFI annually and food insecurity rates are a component of Canada’s Official Poverty Dashboard of Indicators^(42)^, governments have yet to set a public policy goal of HFI reduction with a target rate. The lack of a specific public policy objective related to HFI may be impeding deliberate public policy work to reduce rates through the interventions reviewed here or other means.

## Conclusion

This SR examined the existing body of research on public policy interventions to reduce HFI and placed moderate to high certainty on the evidence showing that income supplementation reduces HFI. Many questions remain in terms of how to optimise this intervention, such as the amount, frequency and delivery mechanism of the income supplementation. In addition, no studies have been designed to clearly differentiate interventions that mitigate households’ experiences of food insecurity from those that prevent HFI in the first place.

## Supporting information

Idzerda et al. supplementary material 1 Idzerda et al. supplementary material

Idzerda et al. supplementary material 2 Idzerda et al. supplementary material

Idzerda et al. supplementary material 3 Idzerda et al. supplementary material

Idzerda et al. supplementary material 4 Idzerda et al. supplementary material

Idzerda et al. supplementary material 5 Idzerda et al. supplementary material

## Supplementary material

For supplementary material accompanying this paper visit https://doi.org/10.1017/S1368980024000120
