# Supplementary material for: Public policy interventions to mitigate household food insecurity in Canada: a systematic review
Source: Public Health Nutr. 2024 Jan 15;27(1):e83. doi: 10.1017/S1368980024000120 (PMC10966928; doi:10.1017/S1368980024000120)
Supplement: Idzerda et al. supplementary material 1 — Idzerda et al. supplementary material [file S1368980024000120sup001.docx]

Supplementary Material A: Inclusion and Exclusion Criteria

| **Population:** Households experiencing food insecurity in Canada.  **Intervention(s):** Interventions include both income-based and food-based interventions. Income-based interventions include, but are not limited to social protection programs such as social assistance benefits, universal child care benefits, or cash transfers. Food-based interventions include food banks and community food programs such as cooking classes, debt counselling, community kitchens and community gardening.    *Inclusions*:   1. Interventions that mitigate or prevent household food insecurity will be included, regardless of whether that is the primary aim.   *Exclusions****:***   1. We will not assess the availability of food related to the agricultural supply chain or environmental constraints such as climate change 2. Interventions aimed at improving nutrition outcomes, if there is no measure of household food insecurity. 3. Interventions designed to measure the attitudes and perceptions of the intervention, if there is no measure of household food insecurity.   **Comparator(s)/control:** The nature of the study in question will determine if there is a comparison group, but not all studies will have a comparison group.  *Inclusions:*   1. Studies with comparisons to a control intervention (e.g. usual care, other intervention) and/or no external comparison or control group (e.g., before and after intervention studies where the comparison subjects are the study subjects themselves before an intervention). 2. Studies that evaluate factorial interventions (compare two interventions with each other as well as their interaction). 3. Comparisons to historical records will be included as well as studies that have a participant act as their own control.   *Exclusions*:   1. Prediction and modelling studies will be excluded.   **Outcomes:** Outcomes measuring household food insecurity will be included.  *Inclusions*:   1. Validated food insecurity scales, such as the HFSSM and its derivatives. 2. Self-reported experience or perceptions of household food insecurity.   *Exclusions*:   1. Outcomes designed to capture client satisfaction, attitudes, and perceptions towards the intervention. 2. Caregiver’s perceptions regarding whether the intervention reduced food insecurity.   **Timeline**: Study must have been published after January 1, 2000.  **Setting**: Canada. |
| --- |
