# Supplementary material for: Public policy interventions to mitigate household food insecurity in Canada: a systematic review
Source: Public Health Nutr. 2024 Jan 15;27(1):e83. doi: 10.1017/S1368980024000120 (PMC10966928; doi:10.1017/S1368980024000120)
Supplement: Idzerda et al. supplementary material 2 — Idzerda et al. supplementary material [file S1368980024000120sup002.docx]

Supplementary Material B: Search Strategy

**Number of Results**

| Date | Medline | Embase | EconLit | Scopus |
| --- | --- | --- | --- | --- |
| April 22, 2021 | 1128 | 1469 | 89 | 2711 |
| November 3, 2022 | 210 | 261 | 16 | 329 |
| October 5, 2023 | 321 | 352 | 9 | 537 |

Search Strategy April 2021

Database(s): **Embase**1974 to 2021 April 21
Search Strategy:

| **#** | **Searches** | **Results** |
| --- | --- | --- |
| 1 | *food availability/ | 870 |
| 2 | *food security/ | 1656 |
| 3 | *food insecurity/ | 1926 |
| 4 | exp *food assistance/ | 745 |
| 5 | exp *catering service/ | 11485 |
| 6 | (food adj3 (secur* or insecur* or adequate or inadequate or adequac* or inadequac* or worry or worries or access* or stress* or hardship? or sufficien* or insufficien* or availab* or acquir* or acquis* or attain* or procur* or assist* or redistribut* or pantr* or aid? or relief or charit* or poverty or desert? or program*)).tw,kw. | 36942 |
| 7 | (food bank? or foodbank? or community food cent* or community kitchen? or community pantr*).tw,kw. | 416 |
| 8 | *hunger/ | 3080 |
| 9 | (hunger or hungry).tw,kw. | 15350 |
| 10 | or/1-9 | 61360 |
| 11 | exp Canada/ or Canadian/ or Canadian Aboriginal/ or (canada* or canadia* or canadien* or ottawa* or british columbia* or colombie britannique* or vancouver* or alberta* or edmonton* or calgar* or saskatchewan* or regina* or saskatoon* or manitoba* or winnipeg* or ontari* or toronto* or quebec* or montreal* or new brunswick* or nouveau brunswick* or fredericton* or nova scotia* or nouvelle ecosse* or halifax* or haligonian* or prince edward island* or ile du prince edouard* or pei or charlottetown* or newfoundland* or terre neuve* or labrador* or nfld or yukon* or whitehorse* or northwest territor* or north west territor* or territoires du nord ouest* or nwt or yellowknife* or nunavut* or iqaluit*).tw,kw. | 356050 |
| 12 | and/10,11 | 1698 |
| 13 | limit 12 to ((english or french) and yr="2000 -Current") | 1469 |

Database(s): **Ovid MEDLINE(R) ALL**1946 to April 21, 2021
Search Strategy:

| **#** | **Searches** | **Results** |
| --- | --- | --- |
| 1 | exp *Food Supply/ | 9095 |
| 2 | *Food Assistance/ | 943 |
| 3 | (food adj3 (secur* or insecur* or adequate or inadequate or adequac* or inadequac* or worry or worries or access* or stress* or hardship? or sufficien* or insufficien* or availab* or acquir* or acquis* or attain* or procur* or assist* or redistribut* or pantr* or aid? or relief or charit* or poverty or desert? or program*)).tw,kf. | 31641 |
| 4 | (food bank? or foodbank? or community food cent* or community kitchen? or community pantr*).tw,kw. | 295 |
| 5 | *hunger/ | 2765 |
| 6 | (hunger or hungry).tw,kw. | 11992 |
| 7 | or/1-6 | 47786 |
| 8 | exp Canada/ or exp Indigenous Canadians/ or (canada* or canadia* or canadien* or ottawa* or british columbia* or colombie britannique* or vancouver* or alberta* or edmonton* or calgar* or saskatchewan* or regina* or saskatoon* or manitoba* or winnipeg* or ontari* or toronto* or quebec* or montreal* or new brunswick* or nouveau brunswick* or fredericton* or nova scotia* or nouvelle ecosse* or halifax* or haligonian* or prince edward island* or ile du prince edouard* or pei or charlottetown* or newfoundland* or terre neuve* or labrador* or nfld or yukon* or whitehorse* or northwest territor* or north west territor* or territoires du nord ouest* or nwt or yellowknife* or nunavut* or iqaluit*).tw,kf,kw. | 279285 |
| 9 | and/7-8 | 1247 |
| 10 | limit 10 to (yr="2000 -Current" and (english or french)) | 1128 |

Database(s): **Econlit**1886 to April 15, 2021
Search Strategy:

| **#** | **Searches** | **Results** |
| --- | --- | --- |
| 1 | (food adj3 (secur* or insecur* or adequate or inadequate or adequac* or inadequac* or worry or worries or access* or stress* or hardship? or sufficien* or insufficien* or availab* or acquir* or acquis* or attain* or procur* or assist* or redistribut* or pantr* or aid? or relief or charit* or poverty or desert? or program*)).tw,kw. | 5991 |
| 2 | (food bank? or foodbank? or community food cent* or community kitchen? or community pantr*).tw,kw. | 73 |
| 3 | (hunger or hungry).tw,kw. | 1086 |
| 4 | or/1-3 | 6783 |
| 5 | (canada* or canadia* or canadien* or ottawa* or british columbia* or colombie britannique* or vancouver* or alberta* or edmonton* or calgar* or saskatchewan* or regina* or saskatoon* or manitoba* or winnipeg* or ontari* or toronto* or quebec* or montreal* or new brunswick* or nouveau brunswick* or fredericton* or nova scotia* or nouvelle ecosse* or halifax* or haligonian* or prince edward island* or ile du prince edouard* or pei or charlottetown* or newfoundland* or terre neuve* or labrador* or nfld or yukon* or whitehorse* or northwest territor* or north west territor* or territoires du nord ouest* or nwt or yellowknife* or nunavut* or iqaluit*).tw,kw. | 27290 |
| 6 | and/4-5 | 108 |
| 7 | limit 7 to (yr="2000 -Current" and (english or french)) | 89 |

SCOPUS

## **( ( TITLE-ABS-KEY ( *food*  W/3  ( *secur**  OR  *insecur**  OR  *adequate*  OR  *inadequate*  OR  *adequac**  OR  *inadequac**  OR  *worry*  OR  *worries*  OR  *access**  OR  *stress**  OR  *hardship?*  OR  *sufficien**  OR  *insufficien**  OR  *availab**  OR  *acquir**  OR  *acquis**  OR  *attain**  OR  *procur**  OR  *assist**  OR  *redistribut**  OR  *pantr**  OR  *aid?*  OR  *relief*  OR  *charit**  OR  *poverty*  OR  *desert?*  OR  *program** ) ) )  OR  ( TITLE-ABS-KEY ( *"food bank?"*  OR  *foodbank?*  OR  *"community food cent*"*  OR  *"community kitchen?"*  OR  *"community pantr*"* ) )  OR  ( TITLE-ABS-KEY ( *hunger*  OR  *hungry* ) ) )  AND  ( ( TITLE-ABS-KEY ( *canada*  OR  *canadian**  OR  *canadien**  OR  *ottawa*  OR  *"british columbia"*  OR  ( *colombie*  PRE/0  *britannique* )  OR  *vancouver*  OR  *alberta*  OR  *edmonton*  OR  *calgar**  OR  *saskatchewan*  OR  *regina*  OR  *saskatoon*  OR  *manitoba*  OR  *winnipeg*  OR  *ontari**  OR  *toronto*  OR  *quebec*  OR  *quebecois*  OR  *montreal*  OR  ( *new*  PRE/0  *brunswick* )  OR  ( *nouveau*  PRE/0  *brunswick* )  OR  *fredericton*  OR  ( *nova*  PRE/0  *scotia* )  OR  ( *nouvelle*  PRE/0  *ecosse* )  OR  *halifax*  OR  *haligonian*  OR  ( *prince*  PRE/0  *edward*  PRE/0  *island* )  OR  ( *ile*  PRE/2  *prince*  PRE/2  *edouard* )  OR  *pei*  OR  *charlottetown*  OR  *newfoundland*  OR  ( *terre*  PRE/0  *neuve* )  OR  *labrador*  OR  *nfld*  OR  *yukon*  OR  *whitehorse*  OR  ( *northwest*  PRE/0  *territor** )  OR  ( *territoires*  PRE/2  *nord*  PRE/2  *ouest* )  OR  *nwt*  OR  *yellowknife*  OR  *nunavut*  OR  *iqaluit* ) ) )  AND  PUBYEAR  >  *1999*  AND  ( LIMIT-TO ( LANGUAGE ,  *"English"* )  OR  LIMIT-TO ( LANGUAGE ,  *"French"* ) )**

|  | Results | 2711 |
| --- | --- | --- |

**Search Strategy – November 2022**

Medline

Database(s): **Ovid MEDLINE(R) ALL**1946 to November 02, 2022
Search Strategy:

| **#** | **Searches** | **Results** |
| --- | --- | --- |
| 1 | exp *Food Supply/ | 10332 |
| 2 | *Food Assistance/ | 1322 |
| 3 | (food adj3 (secur* or insecur* or adequate or inadequate or adequac* or inadequac* or worry or worries or access* or stress* or hardship? or sufficien* or insufficien* or availab* or acquir* or acquis* or attain* or procur* or assist* or redistribut* or suppl* or pantr* or aid? or relief or charit* or poverty or desert? or program*)).tw,kf. | 52338 |
| 4 | (food bank? or foodbank? or community food cent* or community kitchen? or community pantr*).tw,kw. | 395 |
| 5 | *hunger/ | 2951 |
| 6 | (hunger or hungry).tw,kw. | 13382 |
| 7 | or/1-6 | 68926 |
| 8 | exp Canada/ or exp Indigenous Canadians/ or (canada* or canadia* or canadien* or ottawa* or british columbia* or colombie britannique* or vancouver* or alberta* or edmonton* or calgar* or saskatchewan* or regina* or saskatoon* or manitoba* or winnipeg* or ontari* or toronto* or quebec* or montreal* or new brunswick* or nouveau brunswick* or fredericton* or nova scotia* or nouvelle ecosse* or halifax* or haligonian* or prince edward island* or ile du prince edouard* or pei or charlottetown* or newfoundland* or terre neuve* or labrador* or nfld or yukon* or whitehorse* or northwest territor* or north west territor* or territoires du nord ouest* or nwt or yellowknife* or nunavut* or iqaluit*).tw,kf,kw. | 304533 |
| 9 | and/7-8 | 1708 |
| 10 | ((202109* or 2021 09* or 2021 sep* or 202110* or 2021 10* or 2021 oct* or 202111* or 2021 11* or 2021 nov* or 202112* or 2021 12* or 2021 dec* or 2022*) not ("20210901" or "2021 09 01" or "20210902" or "2021 09 02" or "20210903" or "2021 09 03" or "20210904" or "2021 09 04" or "20210905" or "2021 09 05" or "20210906" or "2021 09 06" or "20210907" or "2021 09 07" or "2021 sep 01" or "2021 sep 02" or "2021 sep 03" or "2021 sep 04" or "2021 sep 05" or "2021 sep 06" or "2021 sep 07")).dt,dp. | 2066478 |
| 11 | and/9-10 | 210 |
| 12 | limit 11 to (english or french) | 210 |

Embase

Database(s): **Embase**1974 to 2022 November 02
Search Strategy:

| **#** | **Searches** | **Results** |
| --- | --- | --- |
| 1 | *food availability/ | 922 |
| 2 | *food security/ | 2037 |
| 3 | *food insecurity/ | 2638 |
| 4 | exp *food assistance/ | 955 |
| 5 | exp *catering service/ | 12096 |
| 6 | (food adj3 (secur* or insecur* or adequate or inadequate or adequac* or inadequac* or worry or worries or access* or stress* or hardship? or sufficien* or insufficien* or availab* or acquir* or acquis* or attain* or procur* or assist* or redistribut* or suppl* or pantr* or aid? or relief or charit* or poverty or desert? or program*)).tw,kw. | 57465 |
| 7 | (food bank? or foodbank? or community food cent* or community kitchen? or community pantr*).tw,kw. | 539 |
| 8 | *hunger/ | 3262 |
| 9 | (hunger or hungry).tw,kw. | 16708 |
| 10 | or/1-9 | 83253 |
| 11 | exp Canada/ or Canadian/ or Canadian Aboriginal/ or (canada* or canadia* or canadien* or ottawa* or british columbia* or colombie britannique* or vancouver* or alberta* or edmonton* or calgar* or saskatchewan* or regina* or saskatoon* or manitoba* or winnipeg* or ontari* or toronto* or quebec* or montreal* or new brunswick* or nouveau brunswick* or fredericton* or nova scotia* or nouvelle ecosse* or halifax* or haligonian* or prince edward island* or ile du prince edouard* or pei or charlottetown* or newfoundland* or terre neuve* or labrador* or nfld or yukon* or whitehorse* or northwest territor* or north west territor* or territoires du nord ouest* or nwt or yellowknife* or nunavut* or iqaluit*).tw,kw. | 388257 |
| 12 | and/10-11 | 2157 |
| 13 | ((202109* or sep 2021 or "08 sep 2021" or "09 sep 2021" or 1* sep 2021 or 2* sep 2021 or 3* sep 2021 or september 2021 or 202110* or oct 2021 or "0* oct 2021" or "1* oct 2021" or "2* oct 2021" or "3* oct 2021" or october 2021 or 202111* or nov 2021 or "0* nov 2021" or "1* nov 2021" or "2* nov 2021" or "3* nov 2021" or november 2021 or 202112* or dec 2021 or "0* dec 2021" or "1* dec 2021" or "2* dec 2021" or "3* dec 2021" or december 2021 or 2022*) not ("20210901" or "01 sep 2021" or "20210902" or "02 sep 2021" or "20210903" or "03 sep 2021" or "20210904" or "04 sep 2021" or "20210905" or "05 sep 2021" or "20210906" or "06 sep 2021" or "20210907" or "07 sep 2021")).dc,dd,dp. | 2754177 |
| 14 | and/12-13 | 262 |
| 15 | limit 14 to (english or french) | 261 |

EconLit

Database(s): **Econlit**1886 to October 20, 2022
Search Strategy:

| **#** | **Searches** | **Results** |
| --- | --- | --- |
| 1 | (food adj3 (secur* or insecur* or adequate or inadequate or adequac* or inadequac* or worry or worries or access* or stress* or hardship? or sufficien* or insufficien* or availab* or acquir* or acquis* or attain* or procur* or assist* or redistribut* or suppl* or pantr* or aid? or relief or charit* or poverty or desert? or program*)).tw,kw. | 8051 |
| 2 | (food bank? or foodbank? or community food cent* or community kitchen? or community pantr*).tw,kw. | 99 |
| 3 | (hunger or hungry).tw,kw. | 1203 |
| 4 | or/1-3 | 8887 |
| 5 | (canada* or canadia* or canadien* or ottawa* or british columbia* or colombie britannique* or vancouver* or alberta* or edmonton* or calgar* or saskatchewan* or regina* or saskatoon* or manitoba* or winnipeg* or ontari* or toronto* or quebec* or montreal* or new brunswick* or nouveau brunswick* or fredericton* or nova scotia* or nouvelle ecosse* or halifax* or haligonian* or prince edward island* or ile du prince edouard* or pei or charlottetown* or newfoundland* or terre neuve* or labrador* or nfld or yukon* or whitehorse* or northwest territor* or north west territor* or territoires du nord ouest* or nwt or yellowknife* or nunavut* or iqaluit*).tw,kw. | 28630 |
| 6 | and/4-5 | 165 |
| 7 | ((202109* or 202110* or 202111* or 202112* or 2022*) not ("20210901" or "20210902" or "20210903" or "20210904" or "20210905" or "20210906" or "20210907")).up,so. | 78204 |
| 8 | and/6-7 | 16 |
| 9 | limit 8 to (english or french) | 16 |

Scopus

**Search date**: November 3, 2022

**Results**: 329

( ( LANGUAGE ( english OR french ) ) AND ( ( TITLE-ABS-KEY ( canada OR canadian* OR canadien* OR ottawa OR "british columbia" OR ( colombie PRE/0 britannique ) OR vancouver OR alberta OR edmonton OR calgar* OR saskatchewan OR regina OR saskatoon OR manitoba OR winnipeg OR ontari* OR toronto OR quebec OR quebecois OR montreal OR ( new PRE/0 brunswick ) OR ( nouveau PRE/0 brunswick ) OR fredericton OR ( nova PRE/0 scotia ) OR ( nouvelle PRE/0 ecosse ) OR halifax OR haligonian OR ( prince PRE/0 edward PRE/0 island ) OR ( ile PRE/2 prince PRE/2 edouard ) OR pei OR charlottetown OR newfoundland OR ( terre PRE/0 neuve ) OR labrador OR nfld OR yukon OR whitehorse OR ( northwest PRE/0 territor* ) OR ( territoires PRE/2 nord PRE/2 ouest ) OR nwt OR yellowknife OR nunavut OR iqaluit ) ) AND ( ( TITLE-ABS-KEY ( food W/3 ( secur* OR insecur* OR adequate OR inadequate OR adequac* OR inadequac* OR worry OR worries OR access* OR stress* OR hardship OR hardships OR sufficien* OR insufficien* OR availab* OR acquir* OR acquis* OR attain* OR procur* OR assist* OR redistribut* OR pantr* OR aid OR relief OR charit* OR poverty OR desert OR deserts OR program* ) ) ) OR ( TITLE-ABS-KEY ( "food bank" OR "food banks" OR foodbank OR foodbanks OR "community food centre*" OR "community food center*" OR "community kitchen" OR "community kitchens" OR "community pantr*" OR hunger OR hungry ) ) ) ) ) AND ( ( PUBDATETXT ( "september 2021" AND NOT ( 1 september 2021 OR 2 september 2021 OR 3 september 2021 OR 4 september 2021 OR 5 september 2021 OR 6 september 2021 OR 7 september 2021 ) ) ) OR ( PUBYEAR > 2021 ) OR ( PUBDATETXT ( "october 2021" OR "november 2021" OR "december 2021" ) ) )

**Guide – Ovid Syntax – Search Fields Operators**

- Unless otherwise stated, search terms are free text terms

| **/** | At the end of a term or phrase, searches for term or phrase as a subject heading (index term). Databases use different subject headings systems (or thesaurus) to index content, for example:  Medline: MeSH (Medical Subject Headings)  Embase: Emtree  PsycINFO: American Psychological Association index |
| --- | --- |
| ***** | "Unlimited" truncation symbol. It substitutes for any number of characters at the end of a term or the root of a term, to retrieve plurals and variant spellings  Before a subject heading, indicates that the subject heading is a main topic of the article |
| **?** | "Wildcard" symbol. It substitutes for one or no characters |
| **.ab** | Abstract field code |
| **ab. /freq=2** | Frequency operator in abstract: Searches for terms that are present at least “n” times in the abstract, for ex: ab. /freq=2 |
| **.kf** | Keyword Heading Word field code: searches for a particular word in the Keyword Heading field .kw |
| **.kw** | Keyword Heading field code: contains keywords provided by authors of the article |
| **.mh** | MeSH (Medical subject heading): Medline medical index term |
| **.mp** | Multipurpose field code – searches in title, abstract, drug trade name (tn) and additional fields |
| **.pt** | Publication type |
| **.ti** | Title field code |
| **.tw** | Text Word field code. In Embase includes title, abstract, and drug trade name (tn) |
| **/ae** | Embase/Medline subheading: Adverse Events [drug subheading] |
| **/ci** | Medline subheading: Chemically Induced [disease subheading] |
| **/si** | Embase subheading: Chemically Induced [disease subheading] |
| **/to** | Embase/Medline subheading: Toxicity [drug subheading] |
| **adj#** | Operator that searches for terms adjacent within # number of words, in any order |
| **exp** | "Explodes" a subject heading by searching for the main subject heading and for all Narrower Terms |

**Search Strategy October 2023**

Medline

Database(s): **Ovid MEDLINE(R) ALL**1946 to 2023 October 04
Search Strategy:

| **#** | **Searches** | **Results** |
| --- | --- | --- |
| 1 | exp *Food Supply/ | 10664 |
| 2 | *Food Assistance/ | 1476 |
| 3 | (food adj3 (secur* or insecur* or adequate or inadequate or adequac* or inadequac* or worry or worries or access* or stress* or hardship? or sufficien* or insufficien* or availab* or acquir* or acquis* or attain* or procur* or assist* or redistribut* or suppl* or pantr* or aid? or relief or charit* or poverty or desert? or program*)).tw,kf. | 58561 |
| 4 | (food bank? or foodbank? or community food cent* or community kitchen? or community pantr*).tw,kw. | 453 |
| 5 | *hunger/ | 3034 |
| 6 | (hunger or hungry).tw,kw. | 14340 |
| 7 | or/1-6 | 76016 |
| 8 | exp Canada/ or exp Indigenous Canadians/ or (canada* or canadia* or canadien* or ottawa* or british columbia* or colombie britannique* or vancouver* or alberta* or edmonton* or calgar* or saskatchewan* or regina* or saskatoon* or manitoba* or winnipeg* or ontari* or toronto* or quebec* or montreal* or new brunswick* or nouveau brunswick* or fredericton* or nova scotia* or nouvelle ecosse* or halifax* or haligonian* or prince edward island* or ile du prince edouard* or pei or charlottetown* or newfoundland* or terre neuve* or labrador* or nfld or yukon* or whitehorse* or northwest territor* or north west territor* or territoires du nord ouest* or nwt or yellowknife* or nunavut* or iqaluit*).tw,kf,kw. | 319754 |
| 9 | and/7-8 | 1866 |
| 10 | limit 9 to yr="2022-Current" | 321 |
| 11 | and/9-10 | 321 |
| 12 | limit 11 to (english or french) | 321 |

Embase

Database(s): **Embase**1974 to 2023 October 04
Search Strategy:

| **#** | **Searches** | **Results** |
| --- | --- | --- |
| 1 | *food availability/ | 966 |
| 2 | *food security/ | 2427 |
| 3 | *food insecurity/ | 3186 |
| 4 | exp *food assistance/ | 1105 |
| 5 | exp *catering service/ | 12496 |
| 6 | (food adj3 (secur* or insecur* or adequate or inadequate or adequac* or inadequac* or worry or worries or access* or stress* or hardship? or sufficien* or insufficien* or availab* or acquir* or acquis* or attain* or procur* or assist* or redistribut* or suppl* or pantr* or aid? or relief or charit* or poverty or desert? or program*)).tw,kw. | 64126 |
| 7 | (food bank? or foodbank? or community food cent* or community kitchen? or community pantr*).tw,kw. | 637 |
| 8 | *hunger/ | 3473 |
| 9 | (hunger or hungry).tw,kw. | 18006 |
| 10 | or/1-9 | 91373 |
| 11 | exp Canada/ or Canadian/ or Canadian Aboriginal/ or (canada* or canadia* or canadien* or ottawa* or british columbia* or colombie britannique* or vancouver* or alberta* or edmonton* or calgar* or saskatchewan* or regina* or saskatoon* or manitoba* or winnipeg* or ontari* or toronto* or quebec* or montreal* or new brunswick* or nouveau brunswick* or fredericton* or nova scotia* or nouvelle ecosse* or halifax* or haligonian* or prince edward island* or ile du prince edouard* or pei or charlottetown* or newfoundland* or terre neuve* or labrador* or nfld or yukon* or whitehorse* or northwest territor* or north west territor* or territoires du nord ouest* or nwt or yellowknife* or nunavut* or iqaluit*).tw,kw. | 410398 |
| 12 | and/10-11 | 2365 |
| 13 | limit 12 to yr="2022-Current" | 353 |
| 14 | and/12-13 | 353 |
| 15 | limit 14 to (english or french) | 352 |

EconLit

Database(s): **Econlit**1886 to September 21, 2023
Search Strategy:

| **#** | **Searches** | **Results** |
| --- | --- | --- |
| 1 | (food adj3 (secur* or insecur* or adequate or inadequate or adequac* or inadequac* or worry or worries or access* or stress* or hardship? or sufficien* or insufficien* or availab* or acquir* or acquis* or attain* or procur* or assist* or redistribut* or suppl* or pantr* or aid? or relief or charit* or poverty or desert? or program*)).tw,kw. | 8557 |
| 2 | (food bank? or foodbank? or community food cent* or community kitchen? or community pantr*).tw,kw. | 116 |
| 3 | (hunger or hungry).tw,kw. | 1260 |
| 4 | or/1-3 | 9427 |
| 5 | (canada* or canadia* or canadien* or ottawa* or british columbia* or colombie britannique* or vancouver* or alberta* or edmonton* or calgar* or saskatchewan* or regina* or saskatoon* or manitoba* or winnipeg* or ontari* or toronto* or quebec* or montreal* or new brunswick* or nouveau brunswick* or fredericton* or nova scotia* or nouvelle ecosse* or halifax* or haligonian* or prince edward island* or ile du prince edouard* or pei or charlottetown* or newfoundland* or terre neuve* or labrador* or nfld or yukon* or whitehorse* or northwest territor* or north west territor* or territoires du nord ouest* or nwt or yellowknife* or nunavut* or iqaluit*).tw,kw. | 29207 |
| 6 | and/4-5 | 170 |
| 7 | limit 6 to yr="2022-Current" | 9 |
| 8 | and/6-7 | 9 |
| 9 | limit 8 to (english or french) | 9 |

Scopus

**Search date**: November 3, 2022

**Results**: 537

( ( LANGUAGE ( english OR french ) ) AND ( ( TITLE-ABS-KEY ( canada OR canadian* OR canadien* OR ottawa OR "british columbia" OR ( colombie PRE/0 britannique ) OR vancouver OR alberta OR edmonton OR calgar* OR saskatchewan OR regina OR saskatoon OR manitoba OR winnipeg OR ontari* OR toronto OR quebec OR quebecois OR montreal OR ( new PRE/0 brunswick ) OR ( nouveau PRE/0 brunswick ) OR fredericton OR ( nova PRE/0 scotia ) OR ( nouvelle PRE/0 ecosse ) OR halifax OR haligonian OR ( prince PRE/0 edward PRE/0 island ) OR ( ile PRE/2 prince PRE/2 edouard ) OR pei OR charlottetown OR newfoundland OR ( terre PRE/0 neuve ) OR labrador OR nfld OR yukon OR whitehorse OR ( northwest PRE/0 territor* ) OR ( territoires PRE/2 nord PRE/2 ouest ) OR nwt OR yellowknife OR nunavut OR iqaluit ) ) AND ( ( TITLE-ABS-KEY ( food W/3 ( secur* OR insecur* OR adequate OR inadequate OR adequac* OR inadequac* OR worry OR worries OR access* OR stress* OR hardship OR hardships OR sufficien* OR insufficien* OR availab* OR acquir* OR acquis* OR attain* OR procur* OR assist* OR redistribut* OR pantr* OR aid OR relief OR charit* OR poverty OR desert OR deserts OR program* ) ) ) OR ( TITLE-ABS-KEY ( "food bank" OR "food banks" OR foodbank OR foodbanks OR "community food centre*" OR "community food center*" OR "community kitchen" OR "community kitchens" OR "community pantr*" OR hunger OR hungry ) ) ) ) ) AND ( PUBYEAR > 2021 )
