# Supplementary material for: Public policy interventions to mitigate household food insecurity in Canada: a systematic review
Source: Public Health Nutr. 2024 Jan 15;27(1):e83. doi: 10.1017/S1368980024000120 (PMC10966928; doi:10.1017/S1368980024000120)
Supplement: Idzerda et al. supplementary material 4 — Idzerda et al. supplementary material [file S1368980024000120sup004.docx]

Supplementary Material D: GRADE Decision Rules

| Domain | Judgment | Scoring | Criteria |
| --- | --- | --- | --- |
| Risk of Bias | No serious risk of bias | 0 | - Most studies were considered at low RoB; those that pose a greater risk of bias contribute minimally to the outcome of interest. |
|  | Moderate risk of bias | –0.5 points | - at least one study that contributes significantly to the magnitude of effect is considered to be at moderate risk of bias. |
|  |  | –0.5 point | *If data is synthesized narratively:*   - ≥1 study was at high ROB, but a study at high ROB *was not* responsible for either the highest or lowest or largest effect estimate in the range of estimates for the outcome   *If data synthesized by random effects MA:*   - ≥1 study (but less than 50% of total studies) was at high ROB |
|  | Very serious risk of bias | –1 points | *If data synthesized narratively:*   - ≥1 study was at high ROB, and a study at high ROB *was* responsible for either the highest or lowest or largest effect estimate in the range of estimates for the outcome   *If data synthesized by random effects MA:*   - 50% or more of studies contributing to an outcome were at high ROB |
| Inconsistency | No serious inconsistency | 0 | *If data synthesized narratively:*   - In the judgment of SMEs, heterogeneity in the outcome (i.e. the direction and magnitude of the effect) was considered expected or acceptable, OR - Heterogeneity could be explained, in terms of direction and magnitude of effect, by *a priori*-determined subgroup analyses   *If data synthesized by random effects MA:*   - There is not a wide variance in the point estimates across studies, and - There is overlap of confidence intervals, which suggest variation is likely due to chance, and - tests of heterogeneity have a high p-value (p >0.05) - The *I*^2^ statistic, which quantifies the proportion of the variation in point estimates due to among-study differences, is low AND the sample sizes are small. If the sample sizes are large, the *I*^2^ will be disregarded*. - <40% may be low - 40-75% may be moderate - 75-100% may be considerable   * *Note: when study sample sizes are large, a relatively small difference in point estimates can yield a large I^2^*  OR   - Heterogeneity could be explained by *a priori*-determined subgroup and analyses, and - Removal of the inconsistent study in a sensitivity analysis does not change the point estimate. |
|  | Serious inconsistency | –0.5 points | *If data synthesized narratively:*   - Heterogeneity could be partially (but not completely) explained by *a priori*-determined subgroup analyses.   *If data synthesized by random effects MA:*   - There is a wide variance in the point estimates across studies, and - There is overlap of confidence intervals, which suggest variation is likely due to chance. - tests of heterogeneity have a high p-value (p >0.05) - The *I*^2^ statistic, which quantifies the proportion of the variation in point estimates due to among-study differences, is moderate*. If the sample sizes are large, the *I*^2^ will be disregarded*. - <40% may be low - 40-75% may be moderate - 75-100% may be considerable   * *Note: when study sample sizes are large, a relatively small difference in point estimates can yield a large I^2^*  OR   - Heterogeneity could be partially (but not completely) explained by *a priori*-determined subgroup analyses, and - Removal of the inconsistent study in a sensitivity analysis does not change the point estimate. |
|  | Very Serious Inconsistency | –1 point | *If data synthesized narratively:*   - Heterogeneity could not be explained by *a priori*-determined subgroup analyses   *If data synthesized by random effects MA:*   - There is a wide variance in the point estimates across studies - Minimal or no overlap of confidence intervals, which suggest variation is more that what one would expect of chance alone - tests of heterogeneity has a low p-value (p <0.05) - *I*^2^ statistic, which quantifies the proportion of the variation in point estimates due to among-study differences, is large *. - <40% may be low - 40-75% may be moderate - 75-100% may be considerable   * *Note: when study sample sizes are large, a relatively small difference in point estimates can yield a large I^2^* |
| Indirectness | No serious indirectness | 0 | - The study populations was representative of the general Canadian population within the provinces or the low-income population in the case of a subgroup analysis. |
|  | Serious indirectness | –0.5 points | - The study/studies contributing to an outcome were moderately representative of the general Canadian population within the provinces or the low-income population in the case of a subgroup analysis. |
|  | Very serious indirectness | -1 point | - The study/studies contributing to an outcome was not representative of the general Canadian population within the provinces or the low-income population in the case of a subgroup analysis.   OR   - Indirect measures were used to assess the outcome of interest |
| Imprecision | No serious imprecision | 0 | - If the optimal information size (OIS) criterion is met, or the sample size is very large (at least 2000)   AND   - the **95% CI excludes no effect** (i.e. CI around OR excludes 1.0). |
|  | Serious imprecision | –0.5 points | - If the OIS *was not* met.   OR   - If OIS criterion is met, and the **95% CI overlaps no effect** (i.e. CI includes OR of 1.0). |
| Publication Bias | No serious risk of publication bias | 0 | Due to the limited number of studies included for each population – intervention – outcome combination, it was not possible to undertake a funnel plot.  The search strategy was very comprehensive.  Given the tight-knit community of researchers in the field of food insecurity in Canada, and due to the extensive consultation that we undertook with these experts, we do not believe that there was publication bias that would ultimately change the results of this systematic review for income supplementation studies. |
|  | Serious risk of publication bias | -0.5 points | To be assessed by SMEs  For the housing and food-based studies, the following should be considered.   - study design (experimental vs. observational) - study size (small studies vs. large studies) - lag bias (early publication of positive results) |
| Dose-response gradient |  | +1 point | The presence of a dose-response gradient has long been recognized as an important criterion for believing a putative cause-effect relationship. The presence of a dose-response gradient may increase our confidence in the findings of observational studies and thereby increase the quality of evidence. |
