# Supplementary material for: Public policy interventions to mitigate household food insecurity in Canada: a systematic review
Source: Public Health Nutr. 2024 Jan 15;27(1):e83. doi: 10.1017/S1368980024000120 (PMC10966928; doi:10.1017/S1368980024000120)
Supplement: Idzerda et al. supplementary material 5 — Idzerda et al. supplementary material [file S1368980024000120sup005.docx]

Supplementary Material E: Risk of Bias Rating

| **Author, Publication Year** | **Selection Bias** | **Study Design** | **Confounders** | **Blinding** | **Data Collection Methods** | **Withdrawals and Drop-outs** | **Intervention Integrity** | **RoB Rating** |
| --- | --- | --- | --- | --- | --- | --- | --- | --- |
| **Income Supplementation** | | | | | | | | |
| Brown, 2019 | ? | ? | + | ? | + | NA | + | + |
| Emery, 2013A | ? | ? | + | ? | + | NA | + | + |
| Emery, 2013B | ? | ? | + | ? | + | NA | + | + |
| Ionescu-Ittu, 2014 | ? | ? | + | ? | + | NA | + | + |
| Li, 2016 | ? | ? | + | ? | + | NA | ? | + |
| Loopstra, 2013 | ? | ? | + | - | + | ? | + | ? |
| Loopstra, 2015 | + | ? | + | ? | + | NA | ? | + |
| McIntyre, 2016 | ? | ? | + | ? | + | NA | + | + |
| Men, 2023A | ? | ? | + | + | + | NA | + | + |
| Men, 2023B | + | ? | + | + | + | NA | + | + |
| Tarasuk, 2019 | ? | ? | + | ? | + | NA | + | + |
| **Housing Interventions** | | | | | | | | |
| Kirkpatrick, 2011 | ? | - | + | ? | + | NA | + | ? |
| Li, 2016 | ? | ? | + | ? | + | NA | ? | + |
| Lachaud, 2020 | ? | + | + | - | + | ? | - | - |
| Loopstra, 2013 | ? | ? | + | - | + | ? | + | ? |
| McIntyre, 2017 | ? | ? | + | ? | + | NA | + | + |
| O’Campo, 2017 | ? | + | + | - | + | ? | - | - |
| Pankratz, 2017 | ? | ? | - | - | + | + | + | - |
| **Food Retail Subsidy Intervention** | | | | | | | | |
| St-Germain, 2019 | ? | ? | + | - | + | NA | - | - |

Green (+): strong component; low risk of bias rating
Yellow (?): moderate component; moderate risk of bias rating
Red (-): weak component; high risk of bias rating
White (NA): risk of bias domain not applicable
